# Functional constraints on the evolution of long butterfly proboscides: lessons from Neotropical skippers (Lepidoptera: Hesperiidae)

**DOI:** 10.1111/jeb.12601

**Published:** 2015-03-31

**Authors:** J A S Bauder, L Morawetz, A D Warren, H W Krenn

**Affiliations:** * Department of Integrative Zoology, University of ViennaVienna, Austria; † Department of Behavioural Physiology and Sociobiology, University of WuerzburgWuerzburg, Germany; ‡ McGuire Center for Lepidoptera and Biodiversity, Florida Museum of Natural History, University of FloridaGainesville, FL, USA

**Keywords:** allometry, body size, Costa Rica, flower handling, insects, morphology, mouthparts, nectar intake rate, suction feeding

## Abstract

Extremely long proboscides are rare among butterflies outside of the Hesperiidae, yet representatives of several genera of skipper butterflies possess proboscides longer than 50 mm. Although extremely elongated mouthparts can be regarded as advantageous adaptations to gain access to nectar in deep-tubed flowers, the scarcity of long-proboscid butterflies is a phenomenon that has not been adequately accounted for. So far, the scarceness was explained by functional costs arising from increased flower handling times caused by decelerated nectar intake rates. However, insects can compensate for the negative influence of a long proboscis through changes in the morphological configuration of the feeding apparatus. Here, we measured nectar intake rates in 34 species representing 21 Hesperiidae genera from a Costa Rican lowland rainforest area to explore the impact of proboscis length, cross-sectional area of the food canal and body size on intake rate. Long-proboscid skippers did not suffer from reduced intake rates due to their large body size and enlarged food canals. In addition, video analyses of the flower-visiting behaviour revealed that suction times increased with proboscis length, suggesting that long-proboscid skippers drink a larger amount of nectar from deep-tubed flowers. Despite these advantages, we showed that functional costs of exaggerated mouthparts exist in terms of longer manipulation times per flower. Finally, we discuss the significance of scaling relationships on the foraging efficiency of butterflies and why some skipper taxa, in particular, have evolved extremely long proboscides.

## Introduction

Nectar is commonly regarded as the world's most ubiquitous food source and therefore favoured by many birds, bats and insects (Nicolson, 2007). These taxa have independently evolved various physiological, morphological and behavioural specializations as adaptations for nectar uptake (Pellmyr, 2002; Muchhala & Thomson, 2009; Johnson & Anderson, 2010; Karolyi *et al*., 2012, 2013). Most conspicuous are elongations of the mouthparts which are often shaped as a proboscis in insects (Krenn *et al*., 2005). Euglossine bees, certain tabanid and nemestrinid flies and some hawk moths have evolved extremely long mouthparts that exceed twice the body length to gain access to long-tubed flowers (Amsel, 1938; Borrell, 2005; Borrell & Krenn, 2006; Pauw *et al*., 2009; Karolyi *et al*., 2012, 2014). However, such extremely long mouthparts are rare among butterflies. The proboscis of most European species is medium sized and measures about two-thirds of the body length (Paulus & Krenn, 1996) and averages about 80% in Neotropical butterflies (Kunte, 2007). However, some remarkably long proboscides have been recorded for Neotropical *Eurybia* butterflies (Riodinidae) and for some Neotropical skipper butterflies (Hesperiidae). In some representatives of these families, the proboscis may exceed twice the body length (Kunte, 2007; Bauder *et al*., 2011, 2013, 2014).

The scarcity of long-proboscid butterflies seems peculiar as they could drink nectar from both short- and long-tubed flowers (Agosta & Janzen, 2005), possibly taking a competitive advantage over short-proboscid butterflies. However, disadvantages of having elongated mouthparts can result in longer flower handling times, as has been recorded for hummingbirds, bumblebees, butterflies and some flies (Hainsworth, 1973; Hainsworth & Wolf, 1976; Inouye, 1980; Harder, 1983; Kunte, 2007; Bauder *et al*., 2011; Karolyi *et al*., 2013). Kunte (2007) observed that the flower handling times of butterflies with longer proboscides were significantly longer than the flower handling times of butterflies with normal sized proboscides. Therefore, he regarded the reduced foraging efficiency, that is harvesting less nectar per time, experienced by long-proboscid butterflies as a functional constraint for evolving extraordinarily long proboscides (Kunte, 2007).

However, flower handling time depends on the time required to enter and leave a flower and the actual time needed to take up nectar (Karolyi *et al*., 2013). In butterflies, manipulation time depends on uncoiling the proboscis spiral and finding an entrance into the flower as well as withdrawing and recoiling the proboscis. The suction time is determined by the nectar intake rate, that is nectar volume flow per time unit. Therefore, increased flower handling times of long-proboscid insects could result from problems with flower manipulation, deceleration of nectar intake or a combination of both.

The energy intake rate during feeding influences foraging efficiency (Wolf *et al*., 1972; Heinrich, 1975; Whitham, 1977; May, 1988) and reproductive fitness (Hainsworth *et al*., 1991). Rapid feeding should therefore be favoured by natural selection (Emlen, 1966; Schoener, 1971; Pyke *et al*., 1977). Nectar feeding through a tubular proboscis is subject to physical laws of fluid dynamics, and both the morphological configuration of the feeding apparatus and nectar viscosity modify the rate of nectar intake (Daniel *et al*., 1989; Kim *et al*., 2011; Lee *et al*., 2014). Biophysical models describe factors influencing the speed of fluid feeding and therefore help to understand the constraints regarding the evolution of extremely long proboscides (Kingsolver & Daniel, 1979, 1995; Lee *et al*., 2014). According to the law of Hagen-Poiseuille, the nectar intake rate of butterflies should increase linearly with increasing pressure difference produced by a suction pump and increase with the radius of the food canal to the exponent four. By contrast, it is expected to decline linearly with escalating proboscis length (Kingsolver & Daniel, 1979, 1995). Therefore, insects must compensate for the negative influence of a long proboscis through changes in the radius of the food canal or the size of the suction pump, or otherwise bear this cost through a decreased intake rate (Borrell, 2007). However, exact measurements of nectar intake rates combined with quantitative morphological data over a variety of butterfly species are lacking, although there are some studies on a few butterfly species and other animals such as euglossine bees, hummingbirds and honeyeaters (Hainsworth, 1973; Kingsolver & Daniel, 1983; May, 1985; Mitchell & Paton, 1990; Molleman *et al*., 2005; Borrell, 2007).

Here, we present an integrative approach combining data obtained from behavioural observations and morphological analyses of Neotropical skipper butterflies which vary widely with regard to proboscis length. Video recordings of skippers foraging in the wild and during standardized feeding experiments help explain whether prolonged flower handling times of long-proboscid butterflies result from decelerated nectar intake rates, prolonged flower manipulation times or both. Alternatively, if both of these behavioural aspects were independent of proboscis length, prolonged flower handling times of long-proboscid butterflies would simply result from taking larger amounts of nectar than short-proboscid butterflies. Furthermore, we aimed to analyse the functional implications of interspecific morphological variation to improve our knowledge on the evolution of insect pollinator communities. Therefore, we differentiated the impact of varying proboscis length, body size and cross-sectional area of the food canal on nectar intake rate across species. Based on these results, we discuss the impact of the scaling relationship of body size and proboscis length on nectar intake rate. Finally, we raise the question why some representatives of Hesperiidae, in particular, evolved an extremely long proboscis.

## Materials and methods

### Species sampling

Sampling of Hesperiidae was carried out in the garden and surroundings of the Tropical Station La Gamba (Costa Rica: Puntarenas, Piedras Blancas National Park, 8 °45′ N, 83 °10′ W; 81 m a.s.l.) in September–October 2012 and January–February 2013. Morphometric measurements and feeding experiments were performed with 113 specimens representing 34 species of Hesperiidae from 21 genera. The sample included 38 female and 75 male skippers. Skippers were collected with a hand net and stored in 70% ethanol after the feeding trials. Classification of taxa follows the current phylogeny of Hesperiidae (Warren *et al*., 2009).

### Measurement of body features

Proboscis length and cross-sectional area of the food canal of each individual were measured to estimate their impact on nectar intake rate. However, measuring the exact size of suction pumps requires time-costly morphological reconstructions (Bauder *et al*., 2013; Karolyi *et al*., 2013) and is not manageable for a large sample size. Instead, we measured body size as a correlate for the size of the suction pump, since body size is known to scale with suction pump size (Karolyi *et al*., 2013).

Body length was measured by pinning the body of each ethanol-preserved specimen in a lateral position to a foam mat. After taking a micrograph of the body, the proboscis of each specimen was separated from the head at its base, uncoiled and fixed on a foam mat using insect pins. Micrographs of the body and the proboscis were taken using a Nikon SMZ 1500 stereomicroscope (Nikon, Tokyo, Japan) equipped with an Optocam-I digital camera (Nikon). Micrographs were imported to ImageJ (U.S. National Institutes of Health, Bethesda USA), and body length as well as proboscis length were measured with the aid of the segmented line tool.

The proboscis was cut off at its base, and the galeae were separated from each other. Subsequently, one galea was mounted onto a microscope slide with the food canal facing upwards, embedded in glycerol and covered with a coverslip. The height of the food canal was measured using a Nikon Eclipse E800 light microscope (Nikon) equipped with a Nikon Fi2-U3 digital camera (Nikon) and the NIS Elements D software (Nikon). The width of the food canal was calculated as the distance in μm between two focal planes situated on the lateral wall of the food canal and on the cuticular spines of the dorsal linkage. We measured the height and width of the food canal in two proboscis regions per galea, located at 10% (proximal) and 80% (distal) of the total proboscis length. We estimated the cross-sectional area of the food canal of a proboscis in approximation to an ellipse and calculated the mean cross-sectional area of the proximal and distal food canal for each proboscis.

### Butterfly feeding experiments

**Figure 1:**
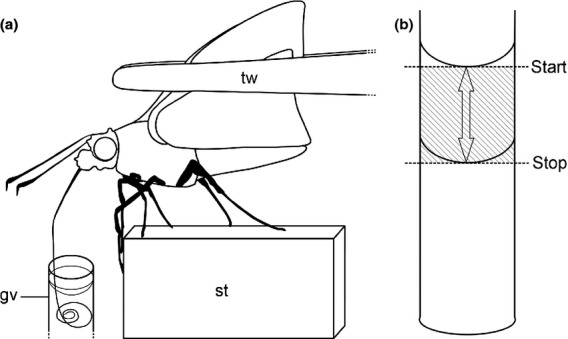
Set-up for video-recorded feeding trials. (a) Skipper feeding from 40% sugar solution. Hungry skippers were locked into position on a stage by pinching the wings together with a pair of tweezers. The proboscis was uncoiled manually and inserted into the glass vial filled with sugar solution. (b) Measuring the ingested volume of sugar solution on video footage. The difference of fluid level from the start and the end of a feeding session was estimated in approximation to a cylinder. gv – glass vial, st – stage, tw – tweezers.

Feeding trials were conducted in an outdoor cage (3 × 2 × 2 m) in the Tropical Research Station, La Gamba, Costa Rica, using skippers that had been caught with a hand net just as they were to start taking nectar from flowers, that is after the proboscis uncoiled. In this way, we ensured that the captured butterflies were hungry and ready to take food which was obligatory for the subsequent feeding trials. Butterflies were stored in a cage until the end of the sampling session which lasted between two and four hours per plant. Feeding experiments were carried out at average ambient air temperatures ranging from 26 to 30  °C. A 40% sugar solution containing sucrose, glucose and fructose, which was prepared in advance and kept refrigerated, was used to imitate the natural nectar of *L. camara* flowers (Alm *et al*., 1990), which are commonly used as a food source by tropical butterflies. Before each feeding trial, the sugar solution was placed under test conditions for half an hour to warm up to ambient air temperature. Each butterfly was immobilized by pinching its wings closed using a pair of tweezers and placed on a feeding platform beside a glass vial (diameter = 3.64 mm) filled with sugar solution (Fig.1a). The proboscis was uncoiled manually with a dissection needle and put into the sugar solution. As soon as the proboscis was inserted into the fluid, the butterfly started to feed. The whole feeding session was recorded with a Sony HDR-XR550VE Handycam (Sony Corporation, Tokyo, Japan). Prior to each feeding session, artificial nectar was renewed to avoid an increase in concentration due to evaporation. Each butterfly was tested once and was subsequently fixed in 70% ethanol.

### Assessment of nectar intake rate

We estimated the ingested volume of sugar solution using images taken from the start and the end of a continuous video-recorded feeding trial using the software PMB 5.0.02.11130 (Sony Corporation). Images were imported to Adobe Photoshop CS4 Extended 11.0.2 (Adobe Systems Incorporated, San Jose, CA, USA), converted to semitransparency and overlaid. In this way, we measured the difference in fluid level with the ruler tool. We estimated the ingested volume in approximation to a cylinder (Fig.1b). Division of the ingested volume of sugar solution by the elapsed time of the nonstop feeding session gave us the rate of volume intake (nL/s).

### Measurement of flower handling time

Skippers were caught from a flower shortly before they would start taking nectar. They were then set free in an outdoor cage equipped with a freshly cut and watered inflorescence of their preferred nectar host plant, that is the plant species that they had visited under natural conditions before being caught (*Stachytarpheta frantzii*:*Autochton longipennis* (*N* = 4), *Urbanus teleus* (*N* = 3), *Morys geisa* (*N* = 2); *Calathea crotalifera*:*Damas clavus* (*N* = 6), *Saliana triangularis* (*N* = 5)). Flower visits were recorded with a Sony HDR-XR550VE Handycam (Sony Corporation). Video recordings were analysed with the software PMB 5.0.02.11130 (Sony Corporation). Behavioural patterns such as proboscis uncoiling, insertion into the floral tube as well as proboscis extraction and recoiling were assessed as manipulation time. In contrast, the period after successful proboscis insertion when the butterfly remained motionless was evaluated as suction time.

### Statistics

All tests were calculated with the statistical package R 3.1.0 (R Development Core Team, 2011). The influence of sex on intake rate was calculated using a general linear model with repeated measurements (random factor: genus) with the function *lme* of the package *nlme* (Pinheiro *et al*., 2014). Correlation between variables was assessed using a Pearson correlation with the function *rcorr* of the package *Hmisc* (Harrell, 2012). The influence of proboscis length and food canal area on intake rate was calculated for a set of 21 genera using the phylogenetic comparative method *comp.gee* as implemented in the package *ape* (Paradis *et al*., 2004). This method accounts for the phylogenetic relationship between genera as genera cannot be regarded as independent from each other. The phylogenetic matrix implemented in the GEE (generalized estimating equation) was constructed using the phylogenetic tree by Warren *et al*. (2009). As information about the genetic distance between the genera is not available, we assumed equal branch lengths of one. The usage of genera as units of analysis was due to the low resolution of the skippers' phylogenetic tree.

## Results

### Scaling of feeding morphology and intake rate

Intake rates of 40% sugar solution were measured in a total of 113 Hesperiidae individuals belonging to 34 species and 21 genera (Table1). Intake rate varied widely between 29.1 ± 3.9 nL per second (*Vehilius stictomenes*, Hesperiinae, *N* = 2) and 1467 nL per second (*Bungalotis quadratum*, Eudaminae, *N* = 1). Intake rate did not differ significantly between sexes (F1, 9 = 1.31, *P* = 0.281). Thus, data of both sexes were pooled for all further analysis.

**Table 1:** Body size, proboscis length, food canal area and nectar intake rate were measured in 113 individual butterflies representing 34 species and 21 genera of skippers (Hesperiidae) from Costa Rica. Note: mean values (± standard deviation) are given whenever more than one individual per species was measured

Species	*N*	Body size [mm]	Proboscis length [mm]	Food canal [μm ²]	Intake rate [nL/s]
Eudaminae					
*Astraptes alardus latia* (evans, 1952)	1	27.0	23.5	5941	483
*Astraptes anaphus anetta* (evans, 1952)	1	23.8	19.5	4477	484
*Autochton longipennis* (plötz, 1886)	3	18.6 (± 0.8)	17.3 (± 1.2)	3343 (± 363)	187 (± 41)
*Autochton zarex* (hübner, 1818)	2	18.8 (± 0.3)	16.3 (±1.5)	3392 (± 40)	174 (± 0.5)
*Bungalotis quadratum quadratum* (sepp, 1845)	1	30.4	39.4	10650	1467
*Cogia calchas* (herrich-schäffer, 1869)	3	15.5 (± 1.1)	12.3 (± 0.6)	2250 (± 195)	110 (± 27)
*Spathilepia clonius* (cramer, 1775)	2	21.9 (± 1.3)	16.8 (± 0.2)	4340 (± 564)	303 (± 48)
*Urbanus procne* (plötz, 1881)	3	19.9 (± 1.1)	15.9 (± 0.1)	3991 (± 853)	234 (± 129)
*Urbanus simplicius* (stoll, 1790)	8	19.9 (± 0.7)	16.5 (± 0.7)	3570 (± 397)	184 (± 78)
*Urbanus tanna* (evans, 1952)	7	20.6 (± 0.8)	16.7 (± 0.3)	3608 (± 487)	252 (± 73)
*Urbanus teleus* (hübner, 1821)	4	19.6 (± 0.9)	16.3 (± 0.6)	3082 (± 386)	168 (± 36)
*Typhedanus undulatus* (hewitson, 1867)	1	16.2	12.4	2725	89
Pyrginae					
Celaenorrhini					
*Celaenorrhinus darius* (evans, 1952)	1	21.1	29.8	3435	136
Pyrrhopygini					
*Mysoria ambigua* (mabille & boullet, 1908)	4	23.2 (± 1.0)	15.3 (0.6)	7270 (± 1462)	387 (± 215)
Hesperiinae					
Clade 113					
*Perichares adela* (hewitson, 1867)	8	23.2 (± 1.5)	44.5 (± 4.9)	5663 (± 1068)	500 (± 249)
*Perichares lotus* (A. butler, 1870)	1	22.8	48.3	5901	425
*Pyrrhopygopsis socrates orasus* (H. druce, 1876)	1	26.1	34.4	6792	544
Calpodini					
*Calpodes ethlius* (stoll, 1782)	4	26.1 (± 0.5)	42.2 (± 1.5)	5509 (± 725)	530 (± 61)
*Saliana esperi esperi* (evans, 1955)	2	18.4 (± 1.7)	35.2 (± 2.2)	3082 (± 318)	174 (± 34)
*Saliana longirostris* (sepp, 1840)	1	26.4	42.7	6023	430
*Saliana salius* (cramer, 1775)	3	23.3 (± 0.6)	47.2 (± 5.7)	5197 (± 691)	199 (± 100)
*Saliana severus* (mabille, 1895)	1	29.6	51.8	8510	747
*Saliana triangularis* (kaye, 1914)	6	21.7 (± 1.3)	41.3 (± 2.5)	4234 (± 812)	174.8 (± 71.4)
*Talides hispa* (evans, 1955)	1	26.0	45.5	8171	349
*Thracides phidon* (cramer, 1779)	1	27.0	42.0	7959	484
Anthoptini					
*Corticea lysias lysias* (plötz, 1883)	1	13.7	14.1	1797	149
Moncini					
*Cymaenes alumna* (A. butler, 1877)	2	13.9 (± 0.6)	16.5 (± 1.5)	1490 (± 220)	65 (± 23)
*Morys geisa* (möschler, 1879)	8	15.0 (± 1.0)	20.1 (± 1.9)	1841 (± 489)	65 (± 19)
*Morys micythus* (godman, 1990)	2	14.9 (± 0.2)	19.6 (± 0.8)	2310 (± 16)	118 (± 5)
*Papias phaeomelas* (hübner, 1831)	10	13.8 (± 0.8)	17.3 (± 1.4)	1499 (± 260)	57 (± 18)
*Papias phainis*godman, 1900	1	13.7	16.2	1311	80
*Papias subcostulata* (herrich-schäffer, 1870)	12	18.1 (± 1.0)	25.5 (± 1.4)	2199 (± 405)	96 (± 30)
*Vehilius stictomenes illudens* (mabille, 1891)	2	12.7 (± 0.6)	13.0 (± 0.01)	1089 (± 34)	29 (± 4)
Hesperiini					
*Pompeius pompeius* (latreille, 1824)	5	16.4 (± 0.7)	15.1 (± 0.3)	2413 (± 283)	120 (± 36)

**Figure 2:**
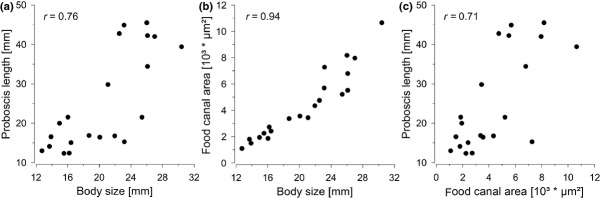
Pearson correlation of all three measured morphological variables suspected to influence intake rate. All variables correlated significantly with each other. (a) Body size and proboscis length (*r* = 0.76). (b) Body size and food canal cross-sectional area (*r* = 0.94). (c) Food canal cross-sectional area and proboscis length (*r* = 0.71). Each data point gives the mean value of one genus of Hesperiidae (*N* = 21).

Proboscis length, food canal area and body size strongly correlated with each other (Figs2a–c). Therefore, it was impossible to include all three measured variables simultaneously into a regression model to evaluate their influence on intake rate. However, increasing proboscis length and food canal area are thought to have contrasting effects on nectar intake rate, which makes the effect of both variables important. On that account, we calculated two separate models with each of these variables. Body size serves as a general description feature for insects and was regarded as the least important variable.

**Figure 3:**
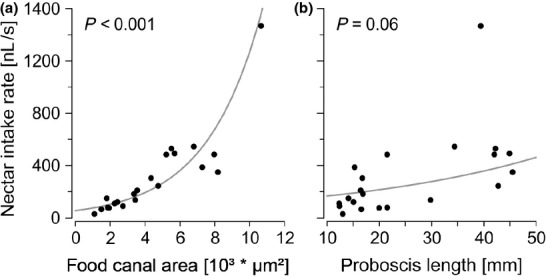
The effect of varying (a) food canal cross-sectional area and (b) proboscis length on nectar intake rate of skipper butterflies. Each data point gives the mean value of one genus of Hesperiidae (*N* = 21). *P* values are from a GEE including a phylogenetic matrix to control for phylogenetic relationship between the measured genera. Bold lines: regression lines.

An increase of food canal area resulted in an increasing intake rate of the tested skipper butterflies (*t*6, 15 = 15.39, *P* = 0.001; Fig.3a). The food canal area is strongly correlated with body size (Fig.2c). This makes it impossible to separate the effect of the food canal area from other features connected with body size (such as suction pump size). In contrast to biophysical models, we found no negative effect of proboscis length on intake rate. Surprisingly, skippers with extremely long proboscides had the tendency to show high intake rates (*t*6, 15 = 2.57, *P* = 0.060; Fig.3b).

### Flower handling time

**Figure 4:**
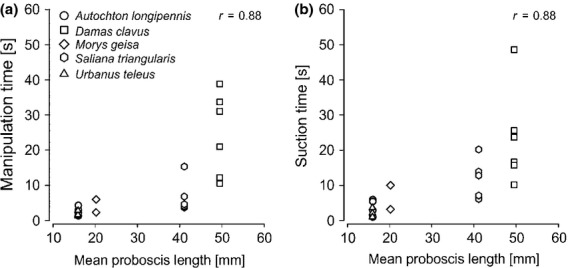
Skippers with long proboscides require more time for flower handling. Both manipulation time (a) and suction time (b) are positively correlated with mean proboscis length.

Flower handling times were measured in five skipper species equipped with medium-sized to extremely long proboscides. Handling time ranged between 6.5 ± 3.2 seconds on shorter tubed *Stachytarpheta* flowers and 48.0 ± 14.2 seconds on the long-tubed *Calathea* flowers. Between 1.9 ± 0.8 and 24.5 ± 11.8 seconds of handling time were spent for proboscis uncoiling, the subsequent search for nectar and recoiling, that is manipulation time, on *Stachytarpheta* and *Calathea* flowers, respectively (Fig.4a). Between 2.2 ± 1.3 and 23.4 ± 13.6 s were spent for actually taking up nectar, that is suction time, on *Stachytarpheta* and *Calathea* flowers, respectively (Fig.4b). Both manipulation time and suction time increase with increasing proboscis length (Fig.4a,b, Spearman correlation: manipulation time *r* = 0.88; suction time *r* = 0.88).

## Discussion

### Functional costs of long proboscides?

Our analysis of nectar intake rates showed that long-proboscid skippers had higher intake rates than short-proboscid skippers. Furthermore, our results confirmed theoretical models by certifying that the food canal area is a crucial factor influencing intake rates of nectar-feeding insects. By contrast, proboscis length did not negatively influence nectar intake rate as predicted by biophysical models. Here, we show that long-proboscid Neotropical skipper butterflies feature a combination of morphological adaptations which enable an efficient nectar uptake. The evolution of a long proboscis is closely linked to other morphological traits such as a large body size, which probably enables the development of a large suction pump to overcome nectar flow resistance, as well as an enlarged food canal. Further evidence for morphological adaptations that allow for efficient nectar intake comes from long-proboscid *Eurybia* butterflies (Bauder *et al*., 2013). These butterflies possess larger dilator muscles of the suction pump in relation to the head capsule volume compared to related short-proboscid metalmark species (Bauder *et al*., 2013). These muscles account for the occurrence of a pressure drop to transport fluid into the gut (Eberhard & Krenn, 2005). In addition, *Eurybia* butterflies were also shown to possess relatively large food canals (Bauder *et al*., 2013).

Behavioural analyses of skippers during flower visitation confirmed the results of Kunte (2007) by showing that long-proboscid skippers require a longer time for a flower visit. This proved true despite their ability to take more nectar in a given time than skippers with shorter proboscides. Further, long-proboscid skipper species spent more time drinking nectar from a flower. These findings indicate that skippers with longer proboscides take higher nectar volumes from the deep-tubed flowers of *Calathea crotalifera* than skippers with shorter proboscides from the flowers of *Stachytarpheta frantzii*. As the corolla tube of *Calathea crotalifera* is deeper than that of *Stachytarpheta frantzii*, skippers that visit *Calathea* flowers most likely ingest higher amounts of nectar as flowers with deep corollae are known to secrete more nectar than shorter flowers (Harder, 1985; Harder & Cruzan, 1990). Given that assumption, possessing a long proboscis can be regarded as an advantage because it enables skippers to gain access to highly rewarding flowers.

However, the flower manipulation times of butterflies increased with proboscis length. Long manipulation times can lower the energy intake rate by decreasing the proportion of foraging time devoted to actually imbibing nectar (Heinrich, 1983; May, 1985). Therefore, longer manipulation times could constitute functional costs of long proboscides. Here, we propose two not mutually exclusive explanations for this phenomenon: longer manipulation times of long-proboscid flower visitors may be caused by the difficulty of inserting the long proboscis into a narrow floral tube. This problem may be due to a poor supply of mechano- or chemosensory information, as other long-proboscid butterflies (Riodinidae) are endowed with significantly fewer sensilla on their proboscides than related short-proboscid species (Bauder *et al*., 2011, 2013).

Alternatively, longer manipulation times of long-proboscid skippers could also be due to differences in flower morphology: long-proboscid skippers preferred the deep-tubed flowers of *Calathea crotalifera*, whereas skippers with shorter proboscides visited flowers of *Stachytarpheta frantzii* with shorter floral tubes. It has been shown that bumble bees require more time to learn complex flower designs, such as long floral tubes with concealed nectar, than simple designs (Laverty, 1994). Several studies on the foraging behaviour of butterflies showed that individual experience gained by successive attempts to forage on a flower can shorten flower manipulation time (Lewis, 1986; Kandori & Ohsaki, 1996; Goulson *et al*., 1997). Therefore, learning the floral morphology could serve as an adaptive strategy for increasing the efficiency of nectar collection (Kandori & Ohsaki, 1996). Further, long-proboscid butterflies could compensate for long manipulation times by visiting fewer nectar-rich flowers instead of many flowers with tiny nectar volumes.

### How does scaling of proboscis and body length affect nectar intake rate?

Recently, we studied representatives of several genera of Hesperiidae with extremely long proboscides for the first time (Bauder *et al*., 2014). Extremely long proboscides that are up to 2.4 times longer than the body and measure up to 52.7 mm evolved several times convergently in Hesperiidae subfamilies (Bauder *et al*., 2014). Among butterflies outside of the Hesperiidae, extremely long proboscides that exceed twice the body length and measure up to 49.9 mm evolved only once in representatives of the genus *Eurybia* (Riodinidae). The extreme size of the proboscides of *Eurybia* butterflies and some skipper species is the outcome of an evolutionary shift from the usual proportional, isometric scaling relationship with body size to a disproportional, allometric scaling with body size (Bauder *et al*., 2014).

The general trend of our data collected from long-proboscid Hesperiidae shows that such allometric scaling of proboscis length with body size virtually comes at no costs in terms of decreased nectar intake rates. However, our measurements also show that the highest nectar intake rate (1467 nL/s) is achieved by the only long-proboscid species featuring an almost isometric scaling relationship of proboscis length and body size, *Bungalotis quadratum* (Eudaminae, *N* = 1). The proboscis of *Bungalotis quadratum* only slightly exceeds its body by 1.3 times, in contrast to all other long-proboscid species that are characterized by proboscis lengths ranging from 1.6 to 2.4 times the body length (Bauder *et al*., 2014). This exceptional result suggests that the scaling relationship of body size and proboscis length may influence the nectar intake rate of a butterfly, which can be maximized in a large specimen featuring an isometric scaling relationship. These findings point to the importance of interspecific variation in scaling relationships and its effect on the foraging efficiency of a butterfly.

### Common grounds of long-proboscid butterflies?

All Neotropical skipper taxa known to have an extremely long proboscis are characterized by a large body size compared to short-proboscid skipper taxa (Bauder *et al*., 2014). However, not all large skippers have an extremely long proboscis, indicating that representatives of certain taxa profit from such disproportional mouthpart sizes.

The most widely accepted hypothesis explaining the evolution of extreme morphologies shaped by natural selection states that the adaptive shift from isometric scaling to allometric scaling represents a selective advantage in foraging, that is gaining access to food resources such as highly rewarding, deep-tubed flowers (Darwin, 1862; Johnson & Steiner, 1997; Alexandersson & Johnson, 2002; Johnson *et al*., 2002; Borrell, 2005; Kunte, 2007; Pauw *et al*., 2009; Krenn, 2010).

In the case of some skippers, such as the calpodines (Hesperiinae), long proboscides may have proven to be useful adaptations to their particular habitat – the deep forests of the Neotropics. These butterflies are known to live in shady, forested habitats (Warren *et al*., 2009) where they visit the deep-tubed flowers of *Calathea* plants for nectar uptake (J. A.-S. Bauder, personal observation; Schemske & Horvitz, 1984), which grow in the understorey of the forest (Weber *et al*., 2001). Furthermore, the long-proboscid *Eurybia* species are also known to use the flowers of these plants not only as a nectar source of the adult butterflies, but also as larval food (Schemske & Horvitz, 1984; DeVries, 1997). Horvitz further remarked that some larval Hesperiidae feed on the leaves of *Calathea* species (Schemske & Horvitz, 1984). The larvae of the long-proboscid skipper species analysed in this study feed on several monocotyledons (Janzen & Hallwachs, 2009) that occur in the understory of the forest (Weber *et al*., 2001), including Marantaceae (*Calathea* sp., *Maranta* sp., *Thalia* sp.), Costaceae (*Costus* sp.), Heliconiaceae (*Heliconia* sp.) and Zingiberaceae (*Renealmia* sp.) (Janzen & Hallwachs, 2009). The convergent evolution of long proboscides in Neotropical deep-forest butterfly species would provide these butterflies exclusive access to deep-tubed flowers, which occur in their microhabitat and cannot be exploited by the vast majority of other butterflies with shorter proboscides.

However, gaining access to large amounts of nectar concealed inside deep-tubed flowers with a long proboscis could also serve to fulfil high energy demands of some skipper taxa resulting from (1) high flight speed (Betts & Wootton, 1988; Chai & Srygley, 1990; Dudley, 1990; Dudley & Srygley, 1994; Hall & Willmott, 2000), (2) high wing loading (Betts & Wootton, 1988; Wickman, 1992; Corbet, 2000) combined with high body mass and (3) the necessity to cover long distances to find patches of larval food plants, which occur in low densities, as it was suggested for hawk moths (Miller, 1997).

Although butterflies have taken centre stage in many taxonomic, ecological and anatomical studies and some species even became increasingly used as model organisms for studying evolution, behaviour or physiology, both Hesperiidae and Riodinidae, the only two butterfly families which comprise long-proboscid species, have been largely left behind (DeVries *et al*., 1992; DeVries, 1997; Warren *et al*., 2009). Hesperiidae have received little attention from collectors because of their dull coloration and difficulties with species identification (Holloway *et al*., 2001; Warren *et al*., 2009). Riodinidae are a diverse group of very tiny inconspicuous butterflies that combine all characteristics of a high tropical diversity with extreme rarity and were long treated as peculiar Neotropical members of Lycaenidae (DeVries *et al*., 1992; DeVries, 1997). Further studies will focus on the nutritional ecology and life-history traits of Hesperiidae and Riodinidae to gain insight into the interspecific variation of lifestyles among species with varying proboscis lengths in combination with analyses of the abundance, distribution, flower tube length and nectar composition of flowering plants in a tropical lowland rainforest.
